# Energy/Electron Transfer Switch for Controlling Optical Properties of Silicon Quantum Dots

**DOI:** 10.1038/s41598-018-35201-0

**Published:** 2018-11-20

**Authors:** Mohammed Abdelhameed, Shawkat Aly, Jeremy T. Lant, Xiaoran Zhang, Paul Charpentier

**Affiliations:** 1 Department of Chemical and Biochemical Engineering, Western University, London, Ontario N6A 5B9 Canada; 2 Department of Mechanical and Materials Engineering, Western University, London, Ontario N6A 5B9 Canada; 3 Department of Biochemistry, Western University, London, Ontario N6A 5B9 Canada

## Abstract

The superior optical properties of Silicon Quantum Dots (SQDs) have made them of increasing interest for a variety of biological and opto-electronic applications. The surface functionalization of the SQDs with aromatic ligands plays a key role in controlling their optical properties due to the interaction of the ligands with the electronic wave function of SQDs. However, there is limited reports in literature describing the impact of spacer groups connecting the aromatic chromophore to SQDs on the optical properties of the SQDs. Herein, we report the synthesis of two SQDs assemblies (1.6 nm average diameter) functionalized with perylene-3,4,9,10-tetracarboxylic acid diimide (PDI) chromophore through N-propylurea and propylamine spacers. Depending on the nature of the spacer, the photophysical measurements provide clear evidence for efficient energy and/or electron transfer between the SQDs and PDI. Energy transfer was confirmed to be the operative process when propylurea spacer was used, in which the rate was estimated to be ~2 × 10^9^ s^−1^. On the other hand, the propylamine spacer was found to facilitate electron transfer process within the SQDs assembly. To illustrate functionality, the water soluble SQD-N-propylurea-PDI assembly was proven to be nontoxic and efficient for fluorescent imaging of embryonic kidney HEK293 cells and human bone cancerous U2OS cells.

## Introduction

Silicon Quantum Dots (SQDs) have recently attracted tremendous attention due to their unique optical properties, including wide absorption spectra, excellent stability against photobleaching compared to conventional dyes, and size-dependent tuneable photoluminescence (PL)^1^. The SQDs have advantages over other quantum dots, including cadmium sulfide (CdS), due to their excellent biocompatibility and biodegradability, low toxicity, and the ease of their surface functionalization^2^. Thus, they are ideal candidates for a wide range of potential applications including bioimaging^3^, photodynamic therapy^4^, sensing^5^, photovoltaics^6^, and light-emitting diodes (LEDs)^7^.

In general, the size-tuneable PL of SQDs is assigned to the quantum confinement effect where the PL is blue-shifted when the size of SQDs is more than ~3 nm. However, a deviation from this behaviour was observed for SQDs of size less than ~2 nm when the PL originates from surface relevant states^8^. The surface functionalization would then play a crucial role towards controlling the optical properties of SQDs^9^. It has been shown in previous studies that surface functionalization of SQDs with aromatic ligands helps tune their optical properties including their quantum yield and PL^10,11^. This strategy potentially can be utilized as a means to control the optical properties of SQDs. Moreover, the functionalization of SQDs with organic ligands increases their stability towards oxidation and prevents them from agglomeration and aggregation^12^.

Only a few reports in literature investigated the influence of aromatic fluorophores covalently linked to SQDs and the role of the spacer connecting the fluorophore on the optical properties of SQDs. Interestingly, the utilization of aromatic fluorophores as a capping agent was found to play a key role to control the optical properties of SQDs^10,13–16^.

Several methods have been reported in literature for preparing SQDs including laser pyrolysis^17^, etching of bulk silicon^18^, nonthermal plasma^19^, and preparation in supercritical fluids^20^. In this work, a facile solution-based reduction method has been adopted with minor modifications to prepare SQDs^21^. Triethoxysilane derivatives and sodium citrate dihydrate were utilized as the silicon source and reducing agent, respectively while the synthesis was carried out in the glycerol green solvent at normal pressure and relatively high temperature (180 °C). Glycerol is a green solvent produced as a byproduct of biodiesel production, and with its high boiling point of 290 °C, and 3 available OH groups to help coordinate to the growing nanocrystals, is of interest for solvent engineering of SQDs.

Here, we report the synthesis of SQDs functionalized with perylene-3,4,9,10-tetracarboxylic acid diimide (PDI) through propylamine and N-propylurea spacers to produce Am-SQD-Per and Urea-SQD-Per, respectively. The PDI dye has been chosen for this study due to its excellent properties including high fluorescence quantum efficiency, high thermal and photochemical stability, and ease of processibility as well as scalability^22^. Additionally, the combination of the planar π-system of PDI and other electron withdrawing and donating groups within the system with SQDs could strongly affect the formed electronic interactions through a possible photoinduced energy and/or electron transfer processes. The latter processes are likely to change the optical properties of the assemblies. The products Am-SQD-Per and Urea-SQD-Per were characterized using high-resolution transmission electronic microscopy (HRTEM), Fourier-transform infrared (FTIR) spectroscopy, X-ray photoelectron spectroscopy (XPS), UV-Vis absorption spectroscopy, and steady-state and times-resolved emission spectroscopy.

## Results and Discussion

**Figure 1:**
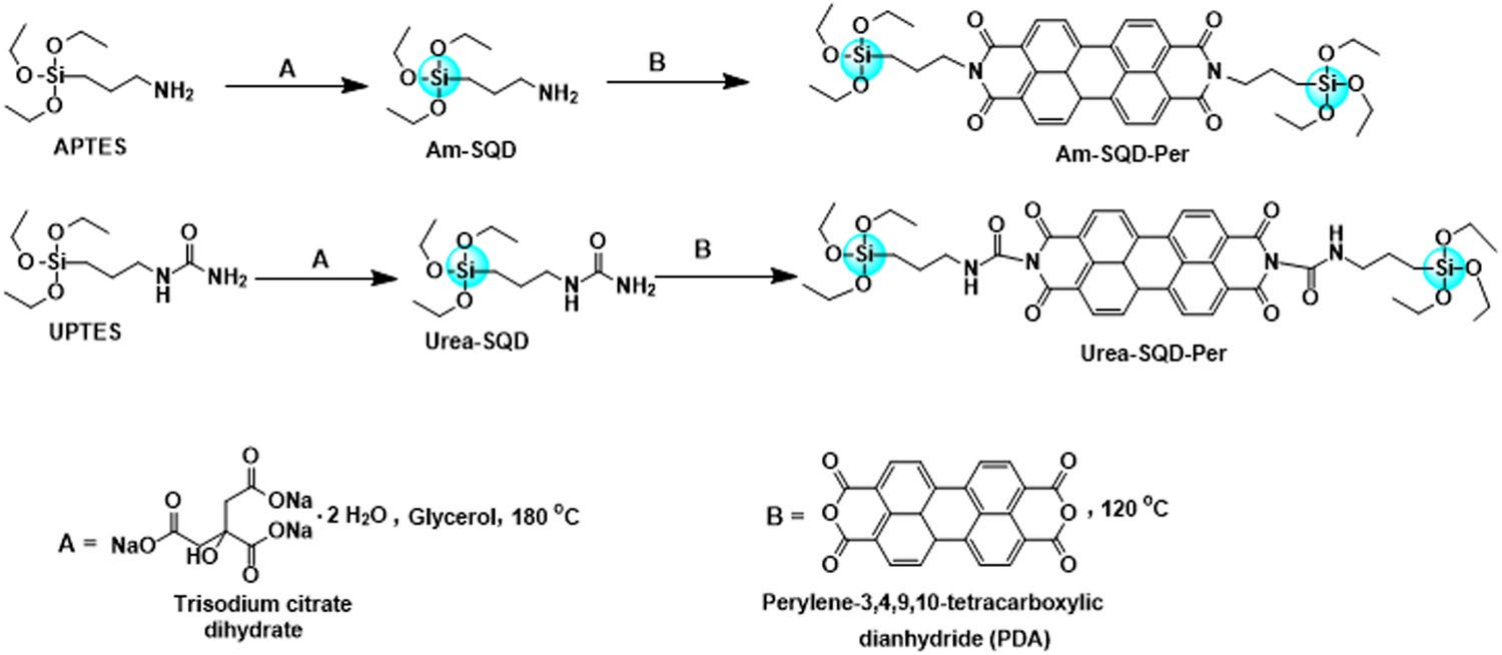
Synthesis route of SQDs (**A**) and their surface functionalization using perylene-3,4,9,10-tetracarboxylic dianhydride (**B**).

The nanoparticles SQDs and their surface functionalization were synthesized as shown in Fig. 1. Both APTES and UPTES were used as the silicon source and were reduced by a citrate reducing agent. This reaction was carried out in glycerol as a high boiling point green solvent under atmospheric pressure and at 180 °C using an oil bath. The resulting SQDs were then functionalized using PDA to produce Am-SQD-Per and Urea-SQD-Per.

### Size and structure

**Figure 2:**
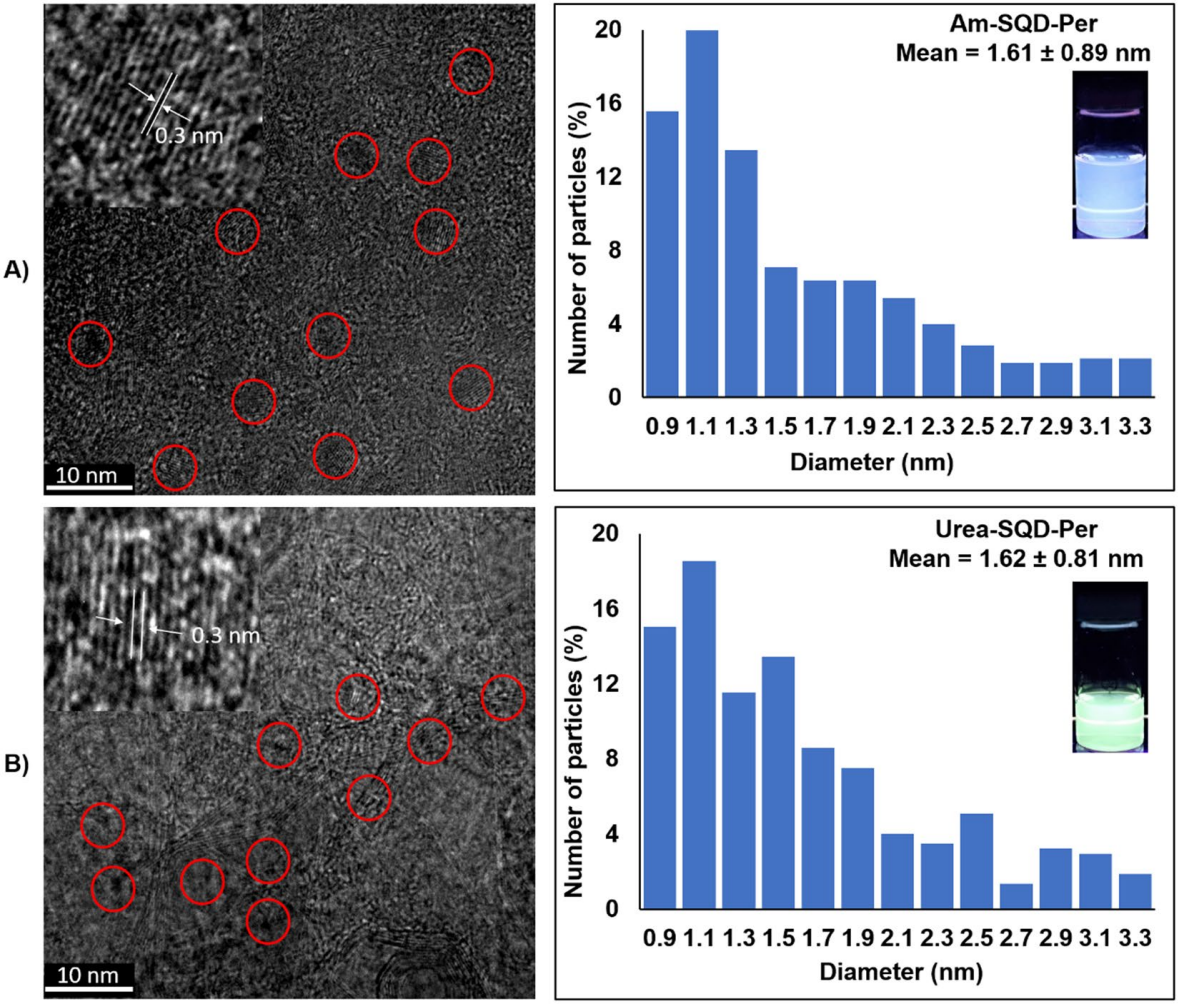
TEM together with HR-TEM (left) and diameter distribution with photographs for solutions under UV (365 nm) irradiation (right) for Am-SQD-Per (**A**) and Urea-SQD-Per (**B**).

Figure 2 shows the TEM, HR-TEM, and size distribution images of the assemblies Am-SQD-Per and Urea-SQD-Per. The TEM images indicate that the functionalized SQDs are quasi-spherical particles with no obvious agglomeration or aggregation. The corresponding size distribution histograms obtained by analyzing of more than 300 dots from different regions of the grids showed that the diameter of these particles ranged from 0.9 to 3.3 nm. The average diameters of SQDs for the compounds Am-SQD-Per and Urea-SQD-Per are 1.61 ± 0.89 and 1.62 ± 0.81 nm, respectively. The functionalized SQDs exhibited high crystallinity which is evidenced by the distinct lattice fringes with 0.30 nm interplanar spacing, as shown in the HR-TEM insets of Fig. 2. This is in agreement with the (111) plane of diamond structured silicon^23^. It should be noted that the low resolution of the TEM and HR-TEM images is assigned to the ultra-small dimensions of these SQDs and the small atomic weight of the silicon atom compared to the counterpart metallic or semiconductor quantum dots, which is known to provide low-quality visualization^10,24^.

**Figure 3:**
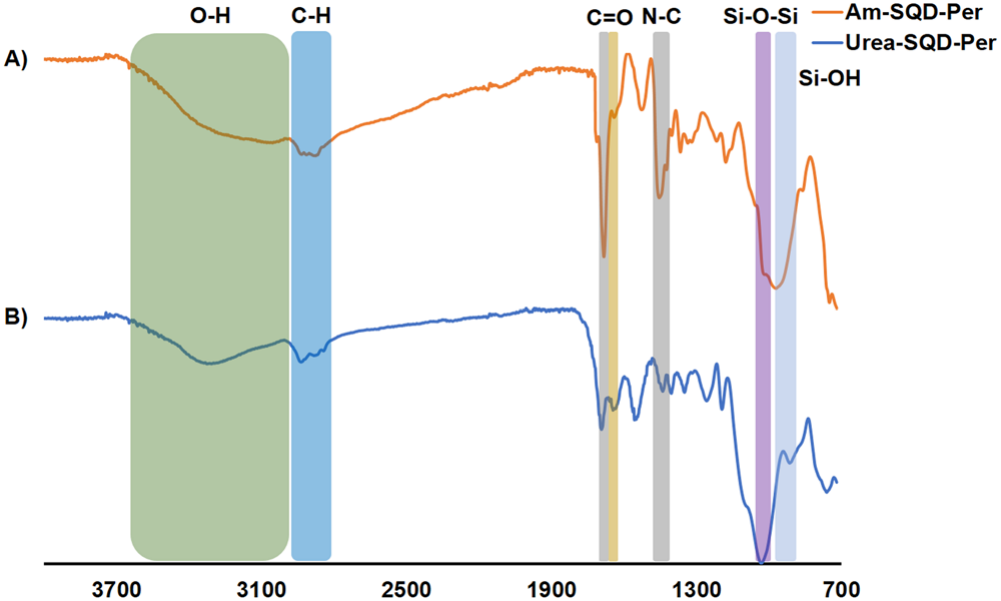
FTIR spectra of Am-SQD-Per (**A**) and Urea-SQD-Per (**B**).

To confirm the attachment of Am-SQD and Urea-SQD to the PDA dye to produce Am-SQD-Per and Urea-SQD-Per, respectively, FTIR and XPS spectroscopy were performed. Figure 3 displays the FTIR spectra of Am-SQD-Per and Urea-SQD-Per. The broad peak at 2980–3660 cm^−1^ can be assigned to the stretching vibration of the O-H bond^25^. The intense peaks at 1019 and 970 cm^−1^ for Am-SQD-Per, and 1024 and 909 cm^−1^ for Urea-SQD-Per, are attributed to Si-O-Si/Si-O-C and Si-OH stretching, respectively^26^. The peaks at 2970–2808 cm^−1^ correspond to the –CH stretching vibrations of the spacer and alkyl group^27^. Both peaks at 1685 and 1638 cm^−1^ for Am-SQD-Per, and 1692 and 1639 cm^−1^ for Urea-SQD-Per, are assigned to imidic C=O stretching (N-C=O)^26^. The peaks at 1439 cm^−1^ in both compounds can be assigned to N-C stretching^28^. Furthermore, the characteristic peaks of the anhydride carbonyl (O-C=O) in the free dye PDA at 1760 and 1720 cm^−1^ are absent in the FTIR spectra of Am-SQD-Per and Urea-SQD-Per. This indicates the success of SQDs binding to the dye.

**Figure 4:**
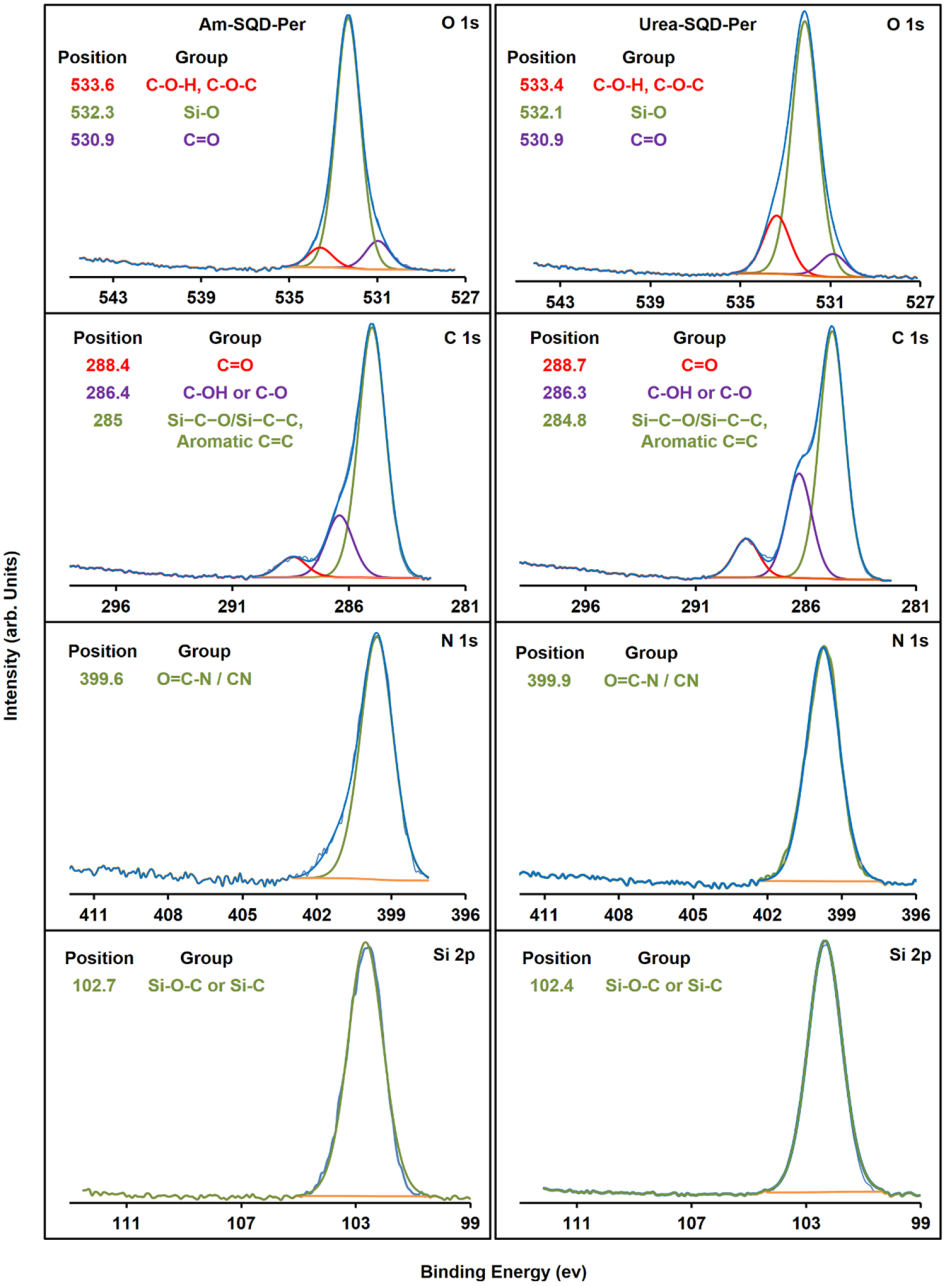
XPS spectra of O 1 s, C 1 s, N 1 s, and Si 2p for Am-SQD-Per and Urea-SQD-Per.

To further confirm the binding of SQDs to the PDA dye, XPS spectroscopy was performed. Figure 4 displays the high resolution XPS spectra of O 1 s, C 1 s, N 1 s, and Si 2p for Am-SQD-Per and Urea-SQD-Per. The deconvoluted peaks of O 1 s appeared at 533.6, 532.3, and 530.9 eV for Am-SQD-Per, and 533.4, 532.1, 530.9 eV for Urea-SQD-Per. These can be assigned to C-O, Si-O, and amidic or imidic carbonyl (-N-C=O), respectively^29–31^. The C 1 s binding energy peaks were present at 288.4, 286.4, and 285 eV for Am-SQD-Per, and 288.7, 286.3, and 284.8 eV for Urea-SQD-Per. These are attributed to C=O of imide or amide bonds (-N-C=O), C-O or C-OH, and Si-C or C=C of PDI kernel, respectively^32–35^. The XPS spectra of N 1 s centered at 399.6 for Am-SQD-Per and 399.9 eV for Urea-SQD-Per signify the presence of N-C or N-C=O^30,36^. The Si 2p peak at 102.7 for Am-SQD-Per and 102.4 eV for Urea-SQD-Per can be assigned to Si-O-C or Si-C^33^. The XPS data are consistent with the FTIR data, providing convincing evidence for successful functionalization of SQDs with PDI.

### Photophysical properties

**Figure 5:**
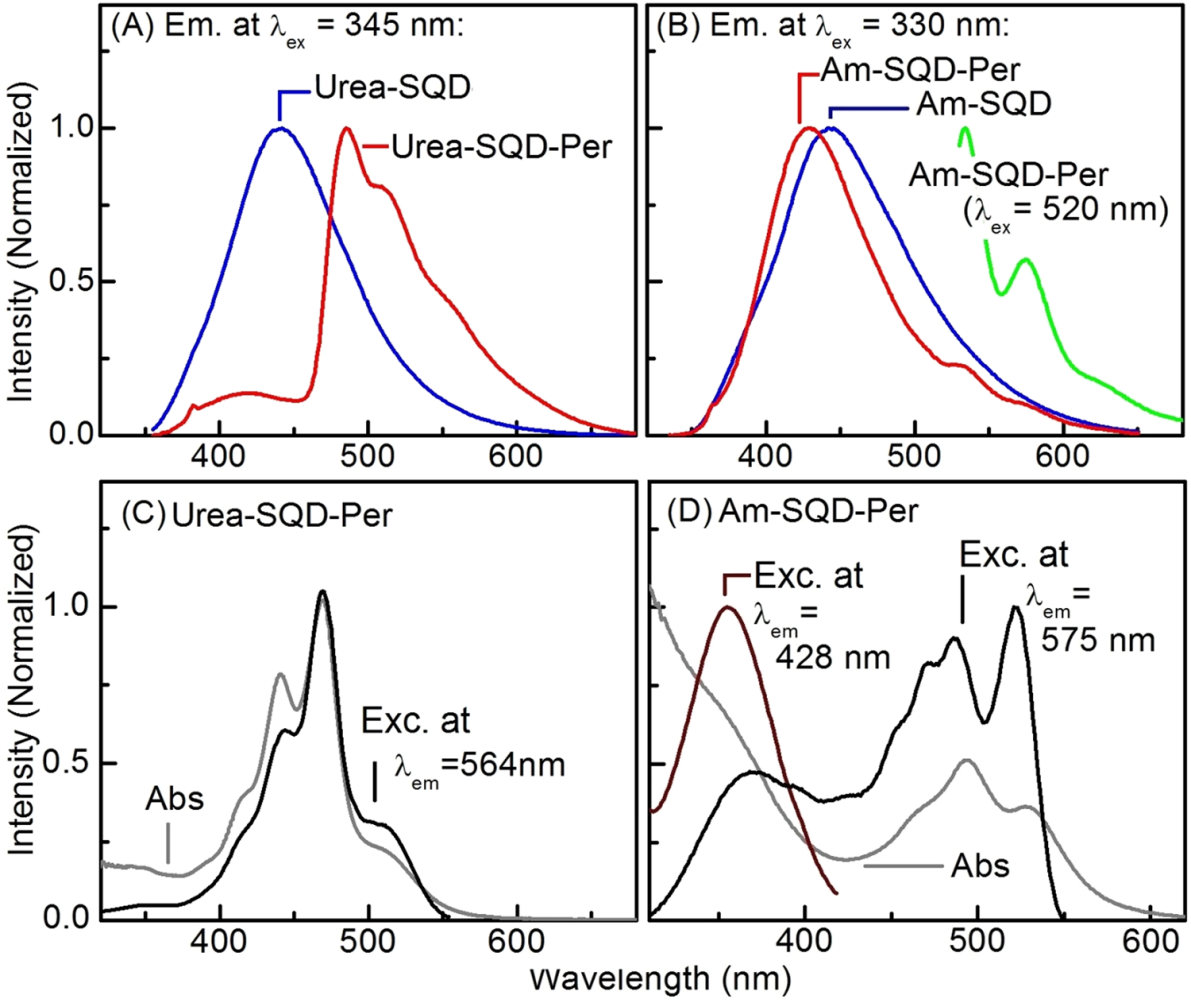
Emission spectra of (**A**) Urea-SQD (blue), Urea-SQD-Per (red) and (**B**) Urea-SQD (blue), Am-SQD-Per (red and green) as well as ground-state absorption (grey) and photoluminescence excitation spectra (black and brown) of (**C**) Urea-SQD-Per and (**D**) Am-SQD-Per collected at room temperature in methanol (excitation wavelength at which emission spectra collected and emission wavelengths at which excitation spectra collected are given on the graph).

Steady-state photoluminescence emission, absorption and excitation spectra for Urea-SQD, Urea-SQD-Per, Am-SQD and Am-SQD-Per in methanol (MeOH) at room temperature are given in Fig. 5(A,B).

Study of the emission spectrum recorded for the starting Urea-SQD (see Fig. 5A) revealed one broad spectrum extended over the spectral range of 350–650 nm, in agreement with reported literature data for amine-terminated silicon quantum dots^37,38^. On the other hand, emission spectrum obtained for Urea-SQD-Per under the same experimental conditions exhibited two main bands (see Fig. 5A). The first emission band showed a resemblance with the starting Urea-SQD with a broad nature over the spectral range between 350–450 nm. An observable blue shift detected in the quantum dot emission in Urea-SQD-Per as compared to the starting Urea-SQD. Considering the average diameter for our SQDs to be 1.6 nm, such shift observed in the emission is likely to be assigned to surface chemistry and independent on quantum confinement^8^. This is in agreement with an earlier report where the emission properties for large QDs (d > ~3 nm) were found to be dominated by quantum confinement while those for smaller size quantum dots (d < ~2 nm) is associated with surface relevant states^8^. In this respect, the observed blue shift should be associated with surface polarity changes due to coupling of the organic dye to the quantum dot surface^23,38–43^. The second band detected in the emission of Urea-SQD-Per came as a relatively stronger band with spectral features extending over 450–650 nm where three main vibronic features detected at 488, 510 and 550 nm. Based on the structured nature of this band and literature data^44^ we assign it to the organic dye, i.e. PDI. From the steady-state emission in Fig. 5A, it is evident that the emission of SQDs is of higher energy compared to that of PDI. An energy level diagram predicted from the lowest energy position of the emission spectra recorded for each of the lumophores constructing the investigated assemblies is given in the supporting information. This energy alignment together with the absorption overlap seen in the ground state absorption allows us to anticipate the possibility of energy transfer. Evidence for the anticipated energy transfer (ET) is found upon examining the PL excitation spectrum collected with emission monochromator fixed at 564 nm where emission is mainly due to the organic dye, (see Fig. 5C). The match found between the absorption and excitation spectra supporting the suggested ET process between the SQDs and the PDI^45^. Further evidences of the assigned ET are given in the time-resolved measurements as discussed below.

Interestingly, different behaviour was found on studying the steady-state emission spectra of Am-SQD-Per in methanol at room temperature (see Fig. 5B). Analysis of the recorded steady-state emission of Am-SQD-Per revealed that spectrum mainly have the signature of SQD emission which suffered from blue shift as compared to its analogue of Am-SQD. Considering the average size of SQD, i.e. 1.6 nm, we confidently assign the observed shift to surface polarity changes associated with the PDI coupling in the same manner to that observed for Urea-SQD-Per^43^. The clear difference to that of Urea-SQD-Per came in the organic dye (PDI) emission vibronics that were absent or suffered from strong quenching in the recorded emission of Am-SQD-Per. Despite this absence or minor contribution of the organic dye in the emission profile, the excitation spectra collected at two different wavelengths revealed the spectral signatures of both the organic dye and the quantum dot; see Fig. 5B. Changing the excitation wavelength to 520 nm, corresponding to the lowest energy (0–0) of the organic dye S_0_-S_1_ as obtained from absorption, allowed us to observe the PDI emission, see Fig. 5B. Being in the same respect, relatively strong quenching in the overall emission was indicated by the calculated PL quantum yield. (see Table 1). This overall photoluminescence quenching together with almost complete disappearance of the organic dye vibronic bands in the emission profile, as compared to its analogues of Urea-SQD-Per, strongly in favourite of photoinduced electron transfer (Pet)^46–51^. Additionally, the excitation spectra collected with emission monochromator fixed at two different wavelengths, one at SQDs emission (428 nm) and the other at PDI emission (575 nm) given in Fig. 5D. Examination of the obtained spectra revealed drastic changes in the relative contribution of the organic dye to the SQDs in the excitation spectrum at 575 nm as compared to their ratio in the absorption spectrum; thus supporting the suggested electron transfer mechanism^51^. Further differences between the two investigated systems of Urea-SQD-Per and Am-SQD-Per were found in the photoluminescence quantum yields calculated under the same experimental conditions; see Table 1.

**Table 1:** Emission quantum yields of SQDs assemblies in methanol at room temperature.

% Ф^a^	Sample			
	Am-SQD	Am-SQD-Per	Urea-SQD	Urea-SQD-Per
total	8.1	2.9	6.3	18.5
SQDs	—	—	—	1.7
Organic dye	—	quenched	—	16.8

^a^Reference used is 6-Aminochrysene and λ_ex_ = 345 nm.

In general, the obtained quantum yield values (which are rather low) are comparable with those recently reported from our group for closely related systems^10^ and in good agreement with literature^41^. While functionalizing SQDs with PDI improved the quantum yield for Urea-SQD-Per as compared to starting Urea-SQD; a large quenching was observed for Am-SQD-Per. The total quantum yield enhancement observed in Urea-SQD-Per can be attributed to the contribution of the organic dye (i.e. PDI, see Fig. 5). On the other hand, the large decrease in quantum yield for Am-SQD-Per argues in favour of the energy loss associated with the suggested electron transfer between SQDs and PDI. Considering this possibility, the photoexcitation energy is anticipated to be lost in formation of the non-luminescent ion pair radical. Indeed, this can help explain the above observation of absence or strong quenching of organic emission in the emission profile displayed in Fig. 5B. Further evidence for the suggested (Pet) found in the time-resolved measurements discussed below.

### Time-resolved single photon counting (TCSPC) measurements

To further decipher the different types of interaction between SQDs and PDI, the synthesized assemblies of Urea-SQD-Per, Am-SQD-Per, and their analogues Urea-SQD and Am-SQD were investigated using time-correlated single photon counting (TCSPC). TCSPC measurements were carried out in the nanosecond (ns) time scale at room temperature. Fluorescence lifetime measurements were collected at maximum emission peaks (λ_em_) as extracted from the steady-state measurements (Table 2). For both assemblies of Urea-SQD and Am-SQD, fluorescence decay traces can be fitted by double exponential functions (see Supporting Information SI5 and SI6). This may indicate the involvement of two competing fast and slow electron–hole recombination processes^52,53^. On the other hand, kinetic traces for Urea-SQD-Per and Am-SQD-Per required equation with three lifetimes for the best fit.

**Table 2:** Fluorescence lifetimes (ns) at room temperature from TCSPC using laser excitation at 375 nm where:

	Am-SQD	Am-SQD-Per	Urea-SQD	Urea-SQD-Per	
λ_em_, nm	450	450	450	445	560
Time, ns (pre)	2.3 ± 0.1 (63)	0.8 ± 0.1 (52)	2.1 ± 0.1 (48)	0.5 ± 0.1 (47)	4.4 ± 0.1
	11.6 ± 0.3 (37)	2.9 ± 0.1 (28)	9.4 ± 0.1 (52)	2.6 ± 0.1 (39)	
		10.6 ± 0.2 (20)		8.9 ± 0.2 (14)	

pre = pre-exponential weighting factor; IRF ≈ 250 ps (from LUDOX SM-30 colloidal silica solution).

**Figure 6:**
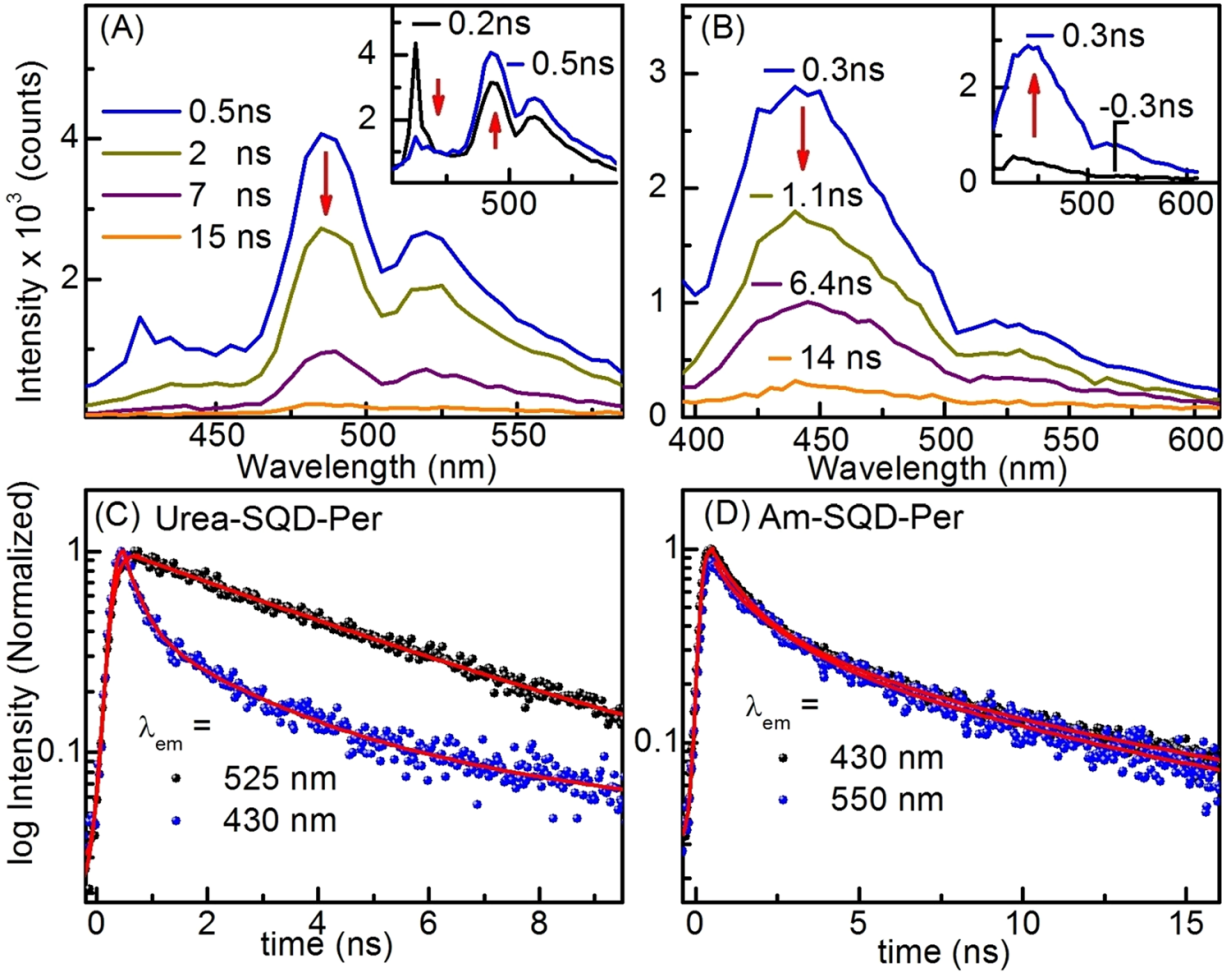
Time-resolved emission spectra of at different delay time of (**A**) Urea-SQD-Per (inset showing early time delay signals) and (**B**) Am-SQD-Per (inset showing early time delay signals) as well as kinetic traces of the decay at two different wavelengths of (**C**) Urea-SQD-Per and (**D**)Am-SQD-Per. Spectra collected using λ_ex_ = 375 nm in methanol at room temperature and delay times are indicated on the graph. (IRF ≈ 250 ps, red lines are fitted curves).

For Urea-SQD-Per, two different kinetic profiles were detected: the first associated with PDI emission and the second corresponding to emission of SQDs. Kinetic traces for Urea-SQD-Per monitored at PDI emission were fit to a mono exponential equation where the extracted lifetime found to be ~4.4 ns. This finding is in good agreement with literature data for PDI-based compunds^50^. Kinetic traces corresponding to SQDs emission maxima revealed three components: one fast together with two of relatively longer lifetimes. The two relatively longer lifetimes are comparable with their analogous Urea-SQD, while the shorter lifetime was found to be in the picosecond (ps) time scale. Time resolved emission spectra (TRES) were done to further investigate the interaction mechanism. TRES collected over the first 500 ps revealed fast decay at emission bands of SQDs almost within the same time domain where those of organic dye are increasing in intensity, see Fig. 6A (inset). Furthermore, as the time delays extended up to 15 ns, an overall decay detected as displayed in Fig. 6A and the kinetic traces in Fig. 6C where the kinetic traces monitored at the two wavelengths corresponding to quantum dot (430 nm) and PDI (525 nm). Kinetic traces are showing fast decay of the quantum dot emission peaks approximately within the same time domain for the rise of the PDI peaks. This rise/decay behavior is perfectly in line with the above suggested photoinduced energy transfer (PET): (Urea-SQD)*-Per → (Urea-SQD)-Per*. Indeed, fitting the kinetic traces showed that Urea-SQD-Per revealed a fast lifetime decay of ~0.5 ns while the lifetime corresponding to the rise of PDI emission found to be ~0.4 ns. Hence, we can estimate the rate of energy transfer to be ~2 × 10^9^ s^−1^; in good agreement with rates estimated for closely related systems in literature^54^.

Another scenario found when studying the behavior of Am-SQD-Per as can be seen in the given spectra displayed in Fig. 6(B,D). TRES studied at the two sides of the laser signal (rise and decay of the laser pulse) are given in Fig. 6B (inset). The emission profile exhibited the signature of the quantum dots regardless of the delay time at which spectrum was collected, which is in line with the strong quenching for the PDI peaks found in the steady-state emission. This observation is in favour of the involvement of a process faster than the temporal resolution of our measurement, i.e. ≤250 ps, responsible for the absence or strong quenching of the PDI emission. These findings together with the above overall weak PL quantum yield values given in Table 1 are indicative of the suggested photoinduced electron transfer process (Pet). Considering the disappearance of the PDI signature within the temporal resolution of our measurements, this allows us to estimate the rate to be ≥4 × 10^9^ s^−1^.

**Figure 7:**
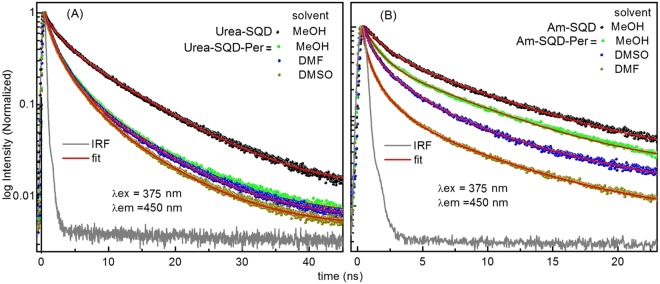
Solvent effect on kinetic traces of (**A**) Urea-SQD-Per and (**B**) Am-SQD-Per. Traces collected using λ_ex_ = 375 nm and emission wavelengths at which traces collected as well as the solvents used are given on graph. (IRF ≈ 250 ps, and red lines are fitted curves).

Confirmation of the charged nature for the interaction involved in Am-SQD-Per was further supported by the kinetic traces collected in different solvents. The charged radical nature of the ion pairs formed due to Pet which makes it sensitive to solvent polarity change (Fig. 7). On the other hand, PET where no charge separation involved solvent change is expected to exhibit a less pronounced impact. Indeed, kinetic traces collected for Urea-SQD-Per revealed minor or no significant differences regardless the solvent used, see Fig. 7. Fluorescence lifetimes extracted from the kinetic traces in different solvents given in Table 3 clearly showed the solvent nature to have a significant impact on the fluorescence kinetic trace for the case of Am-SQD-Per whereas minimum changes detected for that of Urea-SQD-Per. Thus, it is supporting our suggested type of interaction within each of the two systems.

**Table 3:** Solvent effect on fluorescence lifetimes of Am-SQD-Per at room temperature from TCSPC collected using laser excitation (λ_ex_) at 375 nm:

λ_em_, nm	Time, ns (pre)		
	MeOH (PI = 5.1)	DMSO (PI = 7.2)	DMF (PI = 6.4)
450	0.8 ± 0.1 (52)	0.6 ± 0.1 (65)	0.4 ± 0.1 (73)
	2.9 ± 0.1 (28)	2.5 ± 0.1 (26)	1.6 ± 0.1 (21)
	10.6 ± 0.2 (20)	8.5 ± 0.3 (9)	8.3 ± 0.3 (6)
560	0.9 ± 0.1 (45)	0.5 ± 0.1 (57)	0.4 ± 0.1 (66)
	3.1 ± 0.1 (37)	2.2 ± 0.1 (30)	1.7 ± 0.1 (27)
	10.2 ± 0.3 (18)	8.7 ± 0.2 (13)	7.9 ± 0.2 (7

Pre = pre-exponential weighting factor; IRF ≈ 250 ps (from LUDOX SM-30 colloidal silica solution), PI = polarity index.

### Impact of the structure on the nature of the interaction

**Figure 8:**
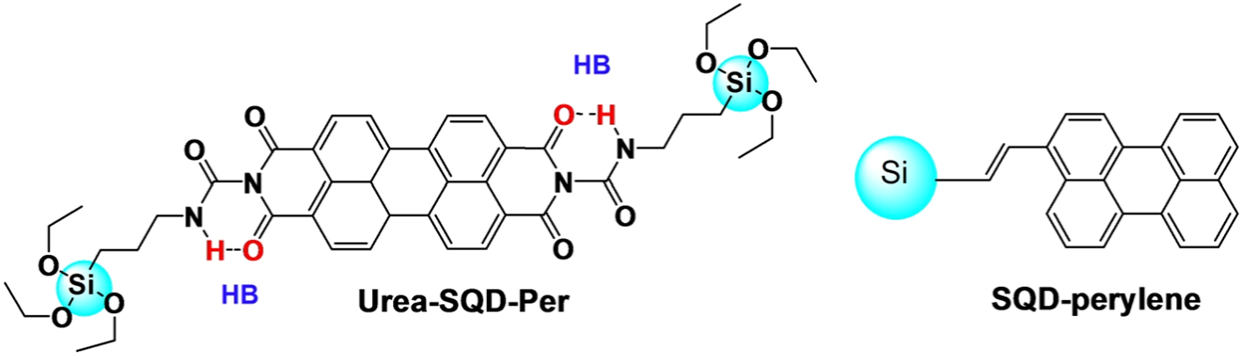
Hydrogen bond (HB) formation through Urea-SQD-Per (left) and earlier investigated SQD-perylene (right) taken from reference^10^.

In an earlier contribution, our group reported an efficient energy transfer to be active between SQDs and perylene dye^10^, see Fig. 8. In this specific system, coupling between the quantum dot and the organic dye achieved using an unsaturated alkyl chain provided an efficient energy transfer between the two luminophores. Herein and in continuation of our interest in tuning the photophysical properties of SQDs based systems, we synthesized two dyad systems built on SQDs and PDI where two different spacers are involved, see Fig. 1. For Am-SQD-Per, with the bridge being a saturated alkyl chain, the two luminophores are expected to have minimum conjugation leaving the electronic system of the two luminophores separated^55^. In this manner; with PDI known to be an electron acceptor^56^, interaction is found to proceed mainly via electron transfer (Pet) as discussed above. It is worthy to indicate here that the possibility of energy transfer interaction cannot be excluded in light of the strong spectral overlap. On the other hand for Urea-SQD-Per, having the hydrogen atom on the amine group closer to the oxygen of the diimide of PDI made hydrogen bond (HB) formation plausible, see Fig. 8. With the PDI redox properties being sensitive to core modification^56^ and energy levels of quantum dots being subtle to modify upon changing the surface chemical structure^57^. Such HB formation possibly rendered electron transfer to be less energetically active. Considering the strong spectral overlap between the absorption of PDI and emission of SQD energy transfer is in turn more likely to be responsible for the observed quenching of the SQD emission. This is supported by the negligible impact of the solvent on the fluorescence lifetime decay curve as discussed in the TSCPC section. Hence, we can pre-design the SQDs assemblies in a way to control the type of interaction permitted throughout and consequently their optical properties.

### Effect of pH change on steady-state emission

**Figure 9:**
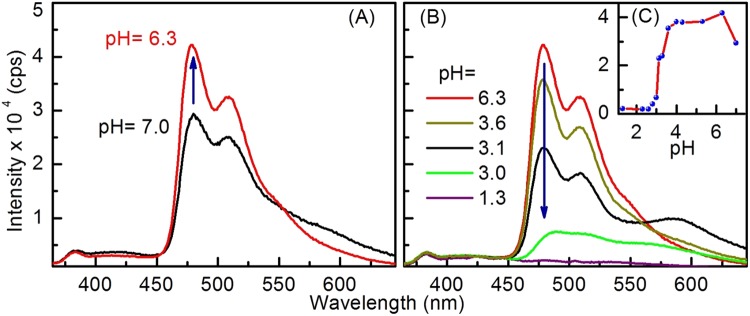
Effect of pH change on the emission of Urea-SQD-Per in water showing initial intensity increase (**A**) intensity decrease (**B**) and emission intensity at 480 nm as function of pH change (**C**).

We extended our study to include the impact of pH changes on synthesized assemblies to better understand the charge-transfer mechanism, as well as the potential for biological applications^58,59^ where pH is an important variable. The study carried out in aqueous solutions of the quantum dots where the pH values were changed while keeping the SQDs concentration constant. The Am-SQD-Per showed negligible changes in emission intensity with changing the pH, see Supporting Information. On the other hand, results revealed a large change in the emission profile of Urea-SQD-Per as function of pH change, see Fig. 9. In general, the pronounced change in intensity detected in the spectral features assigned to PDI, whereas those peaks corresponding to SQD were subject to minor or neglected changes. For the PDI part of the spectrum, first change from the neutral condition to relatively acidic condition resulted in an initial increase of the intensity, see Fig. 9A. Such observed intensity increase with the pH decrease can be attributed to an emission recovery associated with removal charge transfer from the amine group to core of SQDs^60^. In solutions with relatively high pH values, lone pair on the nitrogen atom of the amine group is involved in relaxation processes resulting in a reduced emission. In relatively low pH values, the electron transfer between the amine moieties and the Si core is precluded, yielding higher emission intensity^54^. Further increase of the acid concentration showed a successive decrease in the emission intensity where sharp decrease in the PL intensity collected at 480 nm observed over pH range of 4–2.6, see Fig. 9(B,C). The changes found in the PL part of spectra assigned to the organic dye (i.e. PDI). Such changes can be understood considering that severe changes were earlier reported in literature for PDI emission as function of pH due to stacking or aggregate formation^61^.

### Fluorescent cellular imaging study

**Figure 10:**
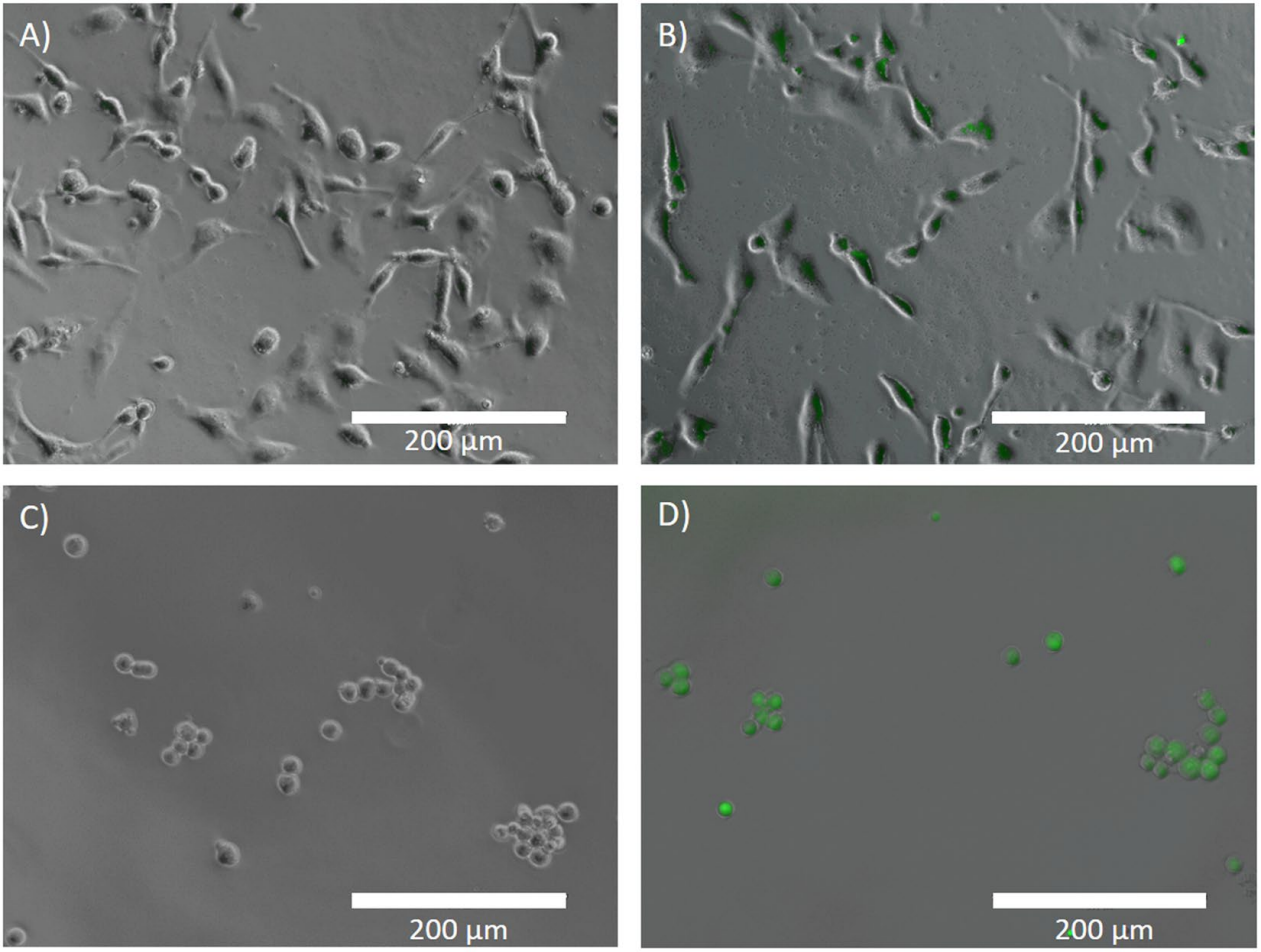
Overlay of the transmission and fluorescence microscope images of the U2OS in the absences of SQDs (**A**) and with functionalized Urea-SQD-Per incorporated inside the cells (**B**) and HEK293 cells with no SQDs (**C**) and with functionalized Urea-SQD-Per (**D**).

To demonstrate the applicability of the Urea-SQD-Per assembly for bioimaging applications, they were utilized for *in vitro* fluorescent imaging of the human osteosarcoma U2OS and human embryonic kidney HEK293 cell lines. Figure 10 shows the U2OS and HEK293 cells that were incubated with Urea-SQD-Per, for which an excitation wavelength of 470 nm was used and the PL at 510 nm was monitored. The control images of both cell lines (Fig. 10A,C) showed no fluorescence from the cells relative to the U2OS and HEK293 cells with the incorporated Urea-SQD-Per (Fig. 10B,D). Thus, the green fluorescence observed in these cells is assigned to the emission from the functionalized SQDs and not autofluorescence from the cells. Therefore, the Urea-SQD-Per assembly are suitable for biological fluorescence imaging applications.

### Impact of Urea-SQD-Per on cellular viability

**Figure 11:**
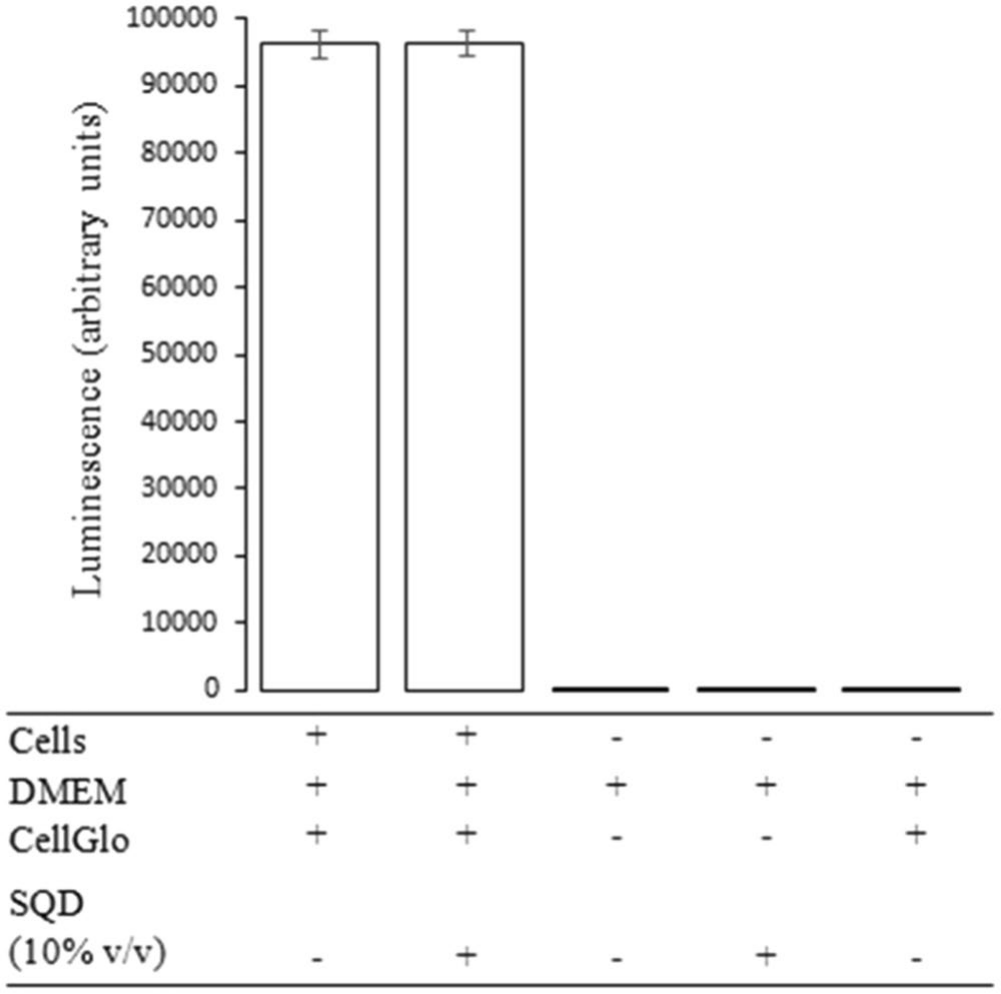
ATP-dependent viability assay. U2OS cells were assayed after treatment with 100 µg/mL urea-SQD-per (SQD) for one hour in high glucose DMEM, following the manufacturers instructions (see methods). Plot shows mean luminescence, which correlates with cellular ATP levels. Errors bars indicate one standard deviation of the mean. Near-undetectable signal from DMEM only, DMEM + SQD, and DMEM + CellGlo reagent indicates the observed luminescence was solely due to detection of cellular ATP.

Using a standard assay, cellular viability was assessed based on the production of ATP in metabolically active cells (see methods). U2OS cells were incubated with and without Urea-SQD-Per at a concentration representing double the concentration used for fluorescent imaging (see Fig. 11), and in six replicates. The viability of cells incubated with Urea-SQD-Per was not significantly different from untreated cells, indicating no detectable cytotoxicity.

## Conclusion

Using simple and environmentally friendly chemistry, we were able to synthesize two dyad systems coupling the SQDs of an average size of ~1.6 nm with perylene-3,4,9,10-tetracarboxylic acid diimide (PDI) chromophore through N-propylurea or propylamine spacers. The chemical nature of the spacer has proven to exert a significant impact on the photophysical properties of the obtained dyad as confirmed by steady state and time-resolved spectroscopy measurements. The results confirmed the possibility to control the nature of the interaction throughout the backbone of the dyad by the change of the spacer used for coupling PDI to the SQDs. While the use of N-propylurea allowed photoinduced energy transfer (PET), the utilization of propylamine permitted photoinduced energy and/or electron transfer within the dyad. Furthermore, PL activity of the synthesized systems was investigated as a function of pH where Am-SQD-Per found to show negligible changes whereas Urea-SQD-Per was sensitive to pH changes. Moreover, *in vitro* fluorescent imaging of the human osteosarcoma U2OS and human embryonic kidney cells HEK293 cell lines showed promising results for bioimaging application.

## Methods and Materials

### Chemicals

(3-Aminopropyl) triethoxysilane (99%, APTES), 1-[3-(trimethoxysilyl)propyl]urea (97%, UPTES), sodium citrate dihydrate (≥99%, citrate), and perylene-3,4,9,10-tetracarboxylic dianhydride (97%, PDA) were used without additional purification. All solvents were dried by passing through MB SPS-800 (MBraun) solvent purification system with water content below 15 ppm.

### Methods

The XPS analyses were carried out with a Kratos Axis Nova spectrometer using a monochromatic Al K(alpha) source (15 mA, 14 kV). The TEM/HRTEM images were recorded using Libra 200 MC operated at 200 kV. The FTIR spectra were measured using a Nicolet 6700 FTIR spectrometer equipped with a smart iTR diamond horizontal attenuated total reflectance (ATR).

The UV–Vis absorption spectra were recorded using a Shimadzu UV-1800 double beam spectrophotometer with a 1 cm path length quartz cuvette. Steady-state emission and excitation spectra were recorded on a Photon Technology International (PTI) spectrofluorometer equipped with a xenon short-arc lamp. All measurements carried out using Felix X32 PTI software for data collection and analysis at 298 K under ambient oxygen in methanol (MeOH).

Time-resolved emission spectra (TRES) and fluorescence lifetimes measurements were carried out using a PicoQuant Fluorescence lifetime system (Picoquant GmbH) equipped with a FluoTime 200 (Fluorescence Lifetime spectrometer), a TimeHarp 200 (Time-correlated Single Photon Counting (TCSPC) system), and a PDL800-B pulsed diode laser driver unit. Samples were excited using a 375 nm using a picoseconds laser diode head (LDH-P-C375). Instrument Response Function (IRF) found to be ~250 picosecond (ps) obtained from analysis of scattered light kinetic trace using LUDOX SM-30 colloidal silica solution at 375 nm. Fluorescence lifetimes were obtained from deconvolution of the kinetic traces of their solutions using global fluorescence decay data analysis software (Fluo Fit) supported by Picoquant GmbH. The TRES was recorded using an automated wavelength scanner and multichannel scaler (MCS) data collection under instrument software control. In this mode, the monochromator was controlled by a stepper motor and automated collection of spectrally resolved lifetime histograms. Data was collected in standard Integration Mode and saved in different blocks of memory for each wavelength. The collected data was then analyzed using FluoPlot software to construct the different emission spectra as function of delay time after excitation with the laser source.

Quatum yield measurements were carried out at 298 K in MeOH using 6-Aminochrysene (Φ_F_ = 18%) as references^62^. Three different solutions for both sample and reference were used for the measurements. Concentrations were adjusted so as to have an absorbance of ~0.05 at the excitation wavelength and absorption spectra recorded five times for accuracy and error minimization.

### Fluorescent imaging

Human U2OS osteosarcoma or HEK293 embryonic kidney cells were grown in 24-well plates in high glucose (4.5 g/L) Dulbecco’s modified Eagle medium containing 3% penicillin and streptomycin and 10% fetal bovine serum (Gibco by Life Technologies). At 60–70% confluency, media was replaced with phosphate buffered saline (1 x PBS pH 7.4; Corning CellGro) and functionalized Urea-SQD-Per dissolved in PBS to a final concentration of 50 ug/mL and incubated for 1–1.5 hr. After incubation, cells were washed twice with PBS to remove excess Urea-SQD-per. Images were captured using an EVOL FL auto fluorescent microscope in GFP mode (excitation/bandwidth = 470/22, emission/bandwidth = 510/42) and phase mode at 20X magnification.

### Cell viability assay

U2OS cells were seeded in equivalent densities in an opaque-walled 96-well plate and grown overnight in high glucose DMEM (4.5 g/L glucose, 10%FBS, 3% penicillin and streptomycin). Cellular viability was determined using a CellTiter-Glo 2.0 Luminescent Cell Viability Assay following the manufacturer’s instructions (Promega). We incubated 6-wells with and without urea-SQD-Per dissolved in PBS to a final concentration of 100 ug/mL for 1 hour. As controls, we measured luminescence of media only, media + SQD (100ug/mL), and media + CellTiter Glo 2.0, each in triplicate. Luminescence readings were collected using a Synergy H1 microplate reader (BioTek).

### Synthesis and purification of SQDs

The SQDs were synthesized using a modified solution-based reduction method^21^. All experiments were performed under argon atmosphere using a schlenk line. In a typical experiment, 0.3 g of citrate was added to 10 ml of warm glycerol with vigorously stirring for 15 minutes till all citrate has been completely dissolved in glycerol. 2 ml of the silicon source (APTES) was then added dropwise to the solution, and the mixture was heated to 180 °C in an oil bath for 3 hours under vigorous stirring. The product (Am-SQD) was then purified using a combination of centrifugation and dialysis against methanol (MWCO of 1 KDa, Spectra/Por® 6 Standard RC Pre-wetted Dialysis Tubing, diameter 29 mm). The previous procedures were repeated using UPTES as the silicon source to produce Urea-SQD.

### Functionalization of SQDs

An excess amount of PDA (2 g) was added to 2 ml of Am-SQD, and the mixture was then heated to 130 °C for 5 hours under argon atmosphere. The product Am-SQD-Per was extracted from the resulting thick red solid by methanol, and insoluble solid reactants were removed by gravity filtration. The filtrate was then concentrated using a rotatory evaporator and purified using dialysis against methanol. Similarly, the compound Urea-SQD-Per was prepared.

## Electronic supplementary material

SUPPLEMENTARY INFORMATION
