# Supplementary material for: Energy/Electron Transfer Switch for Controlling Optical Properties of Silicon Quantum Dots
Source: Sci Rep. 2018 Nov 20;8:17068. doi: 10.1038/s41598-018-35201-0 (PMC6244374; doi:10.1038/s41598-018-35201-0)
Supplement: Supplementary file 1 — SUPPLEMENTARY INFORMATION [file 41598_2018_35201_MOESM1_ESM.docx]

**Energy/Electron Transfer Switch for controlling Optical Properties of Silicon Quantum Dots**

**Supporting Information**

**Mohammed Abdelhameed** *^†a^***, Shawkat Aly** *^†b^***, Jeremy T. Lant** *^c^***, Xiaoran Zhang,** *^a^* **and Paul Charpentier ******^a^*

**
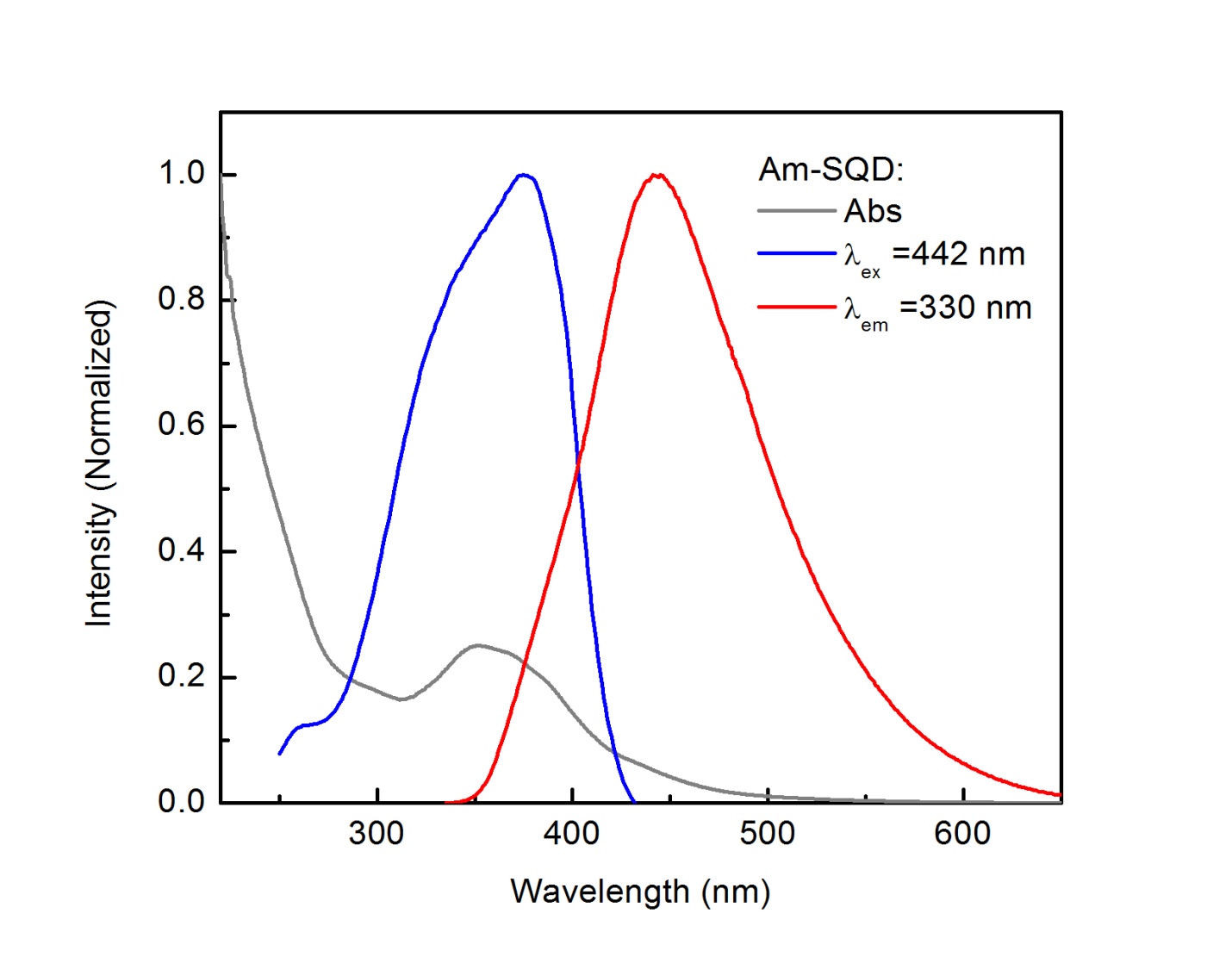
**

SI 1. Absorption, excitation spectra and emission of Am-SQD (A) collected at room temperature in methanol (λ_em_ and λ_ex_ indicated on graph).

**
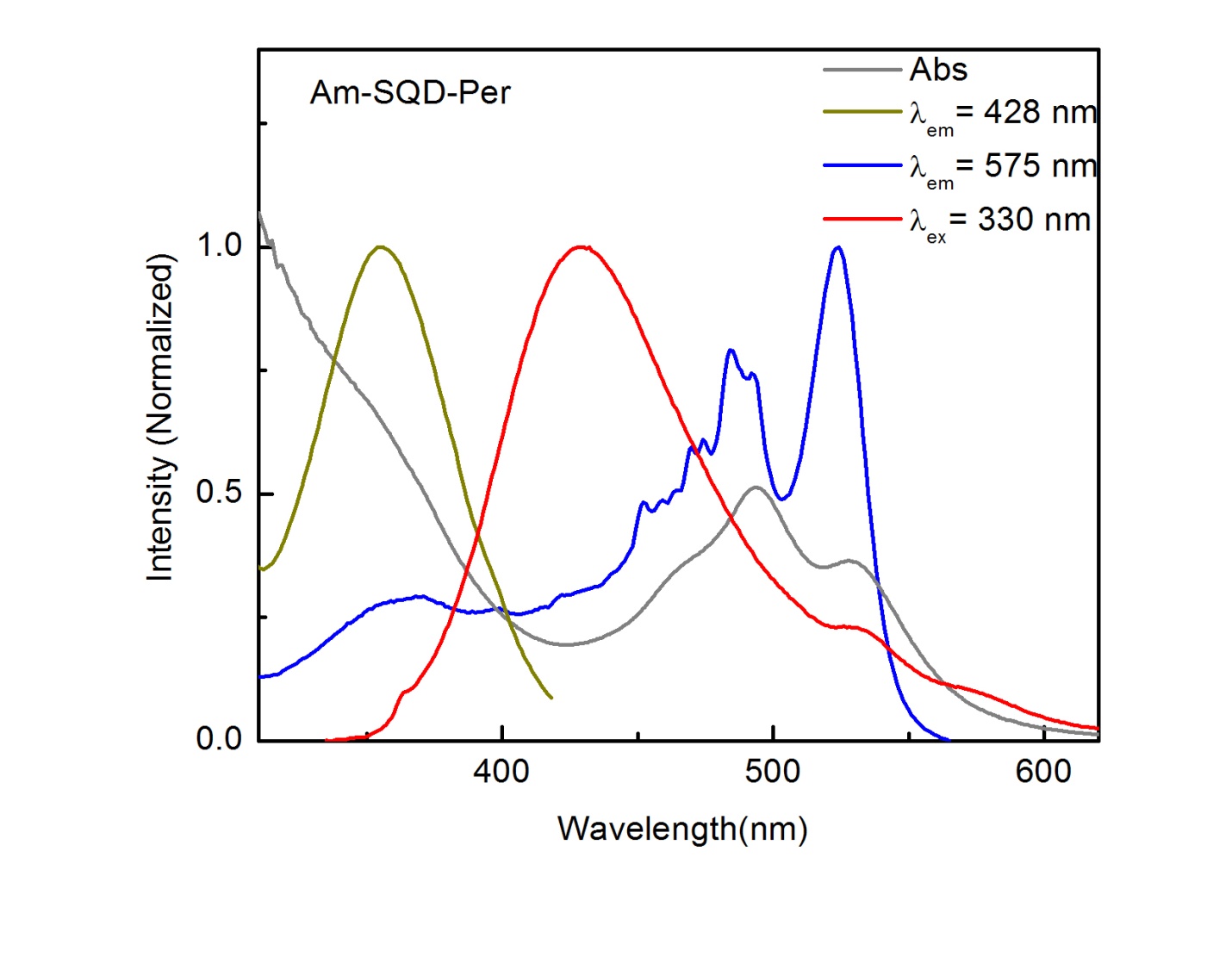
**

SI 2. Absorption, excitation spectra and emission spectra of Am-SQD-Per (A) collected at room temperature in methanol (λ_em_ and λ_ex_ indicated on graph).

**
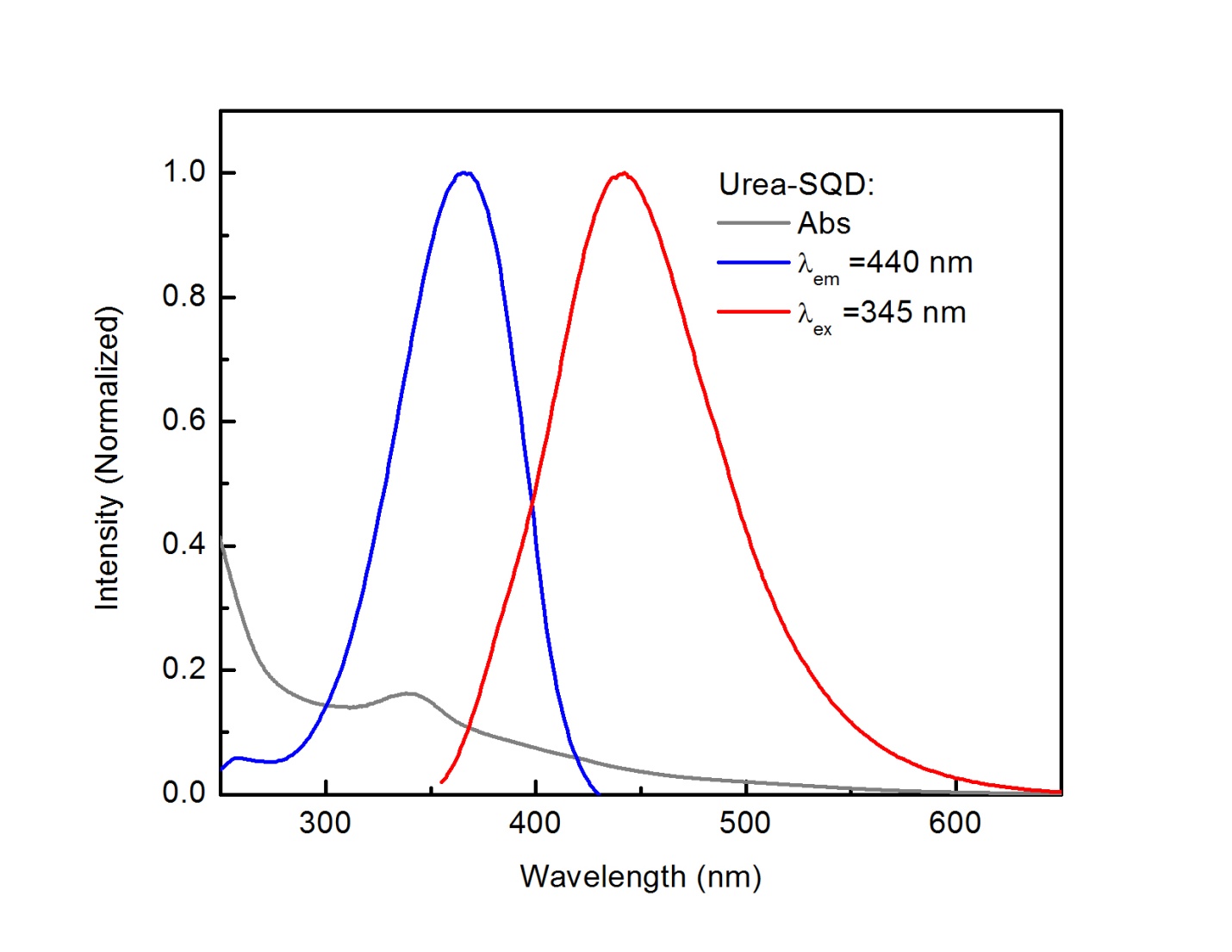
**

SI 3. Absorption, excitation spectra and emission spectra of Urea-SQD (A) collected at room temperature in methanol (λ_em_ and λ_ex_ indicated on graph).

**
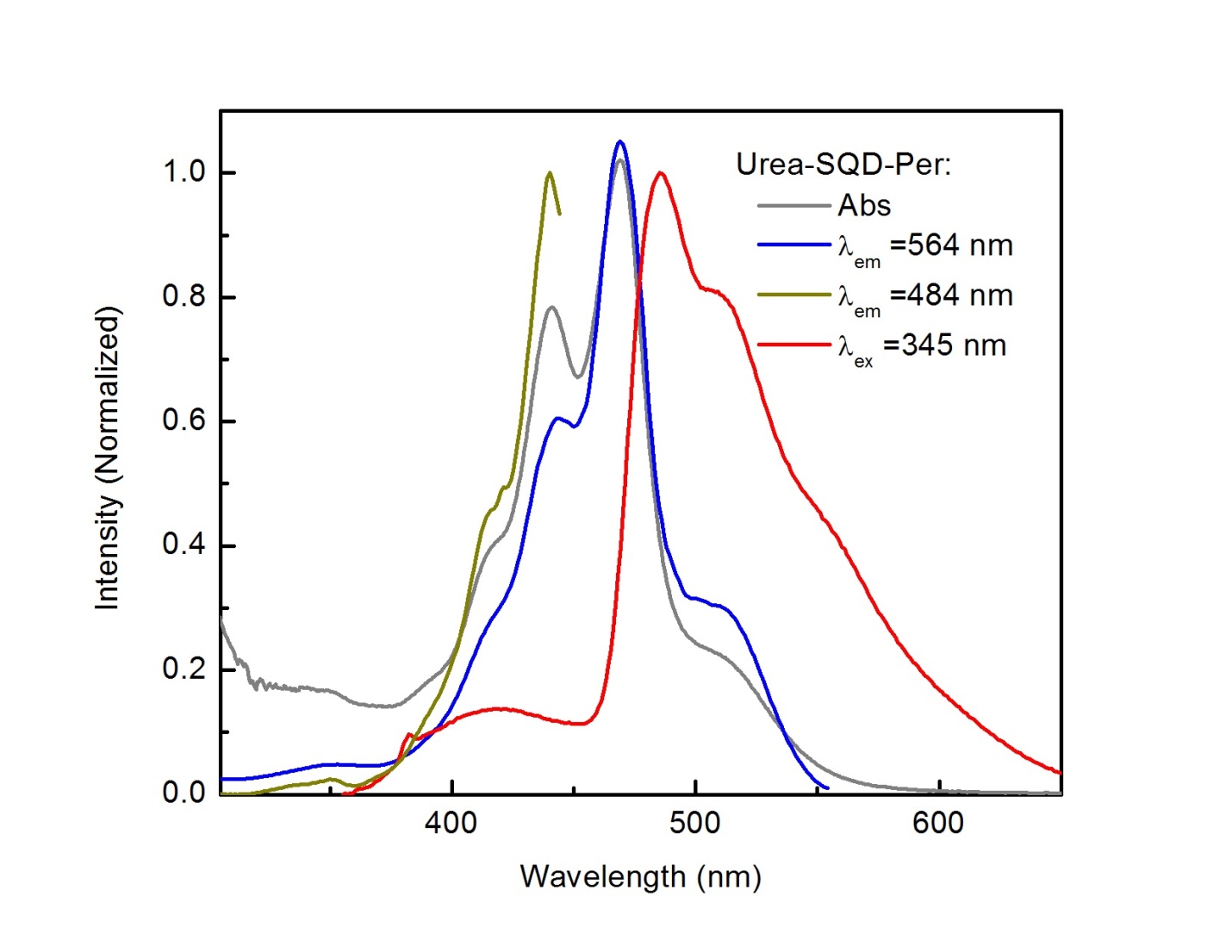
**

SI 4. Absorption, excitation spectra and emission spectra of Urea-SQD-Per (A) collected at room temperature in methanol (λ_em_ and λ_ex_ indicated on graph).

**
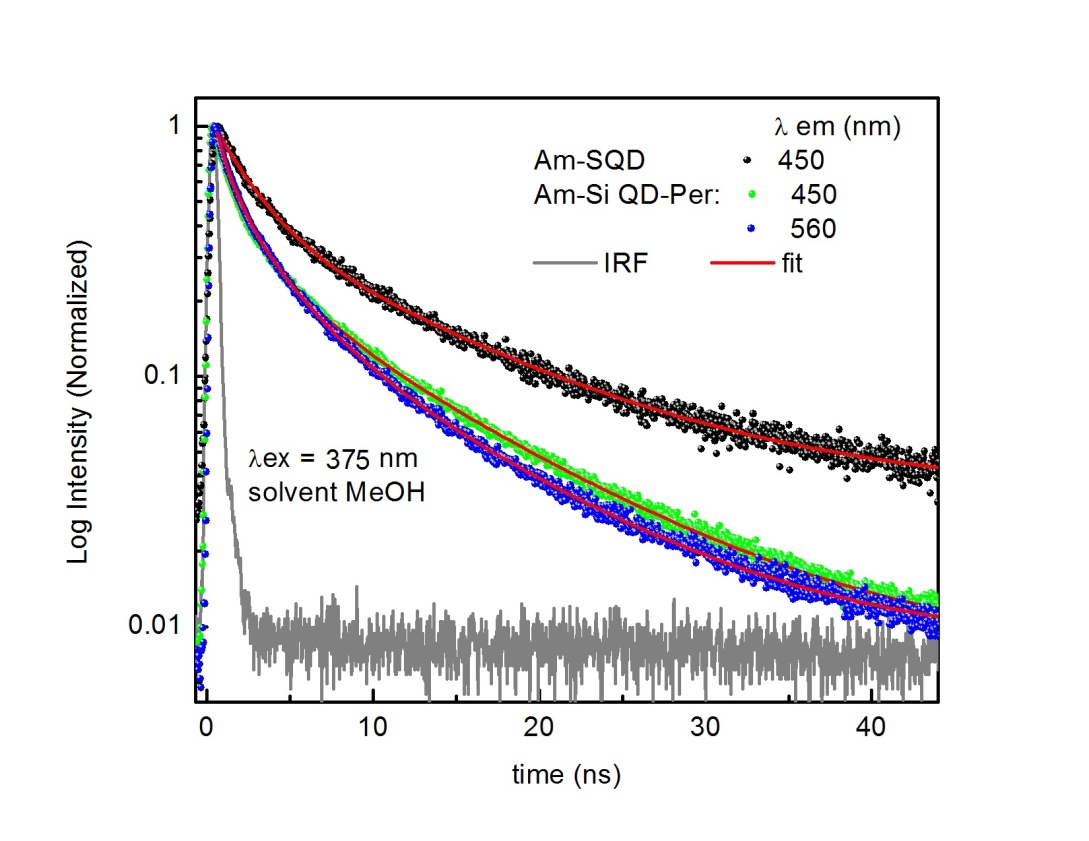
**

SI 5. Kinetic traces of Am-SQD and Am-SQD-Per collected at room temperature in methanol (λ_em_ and λ_ex_ indicated on graph; IRF ≈ 250 ps).

**
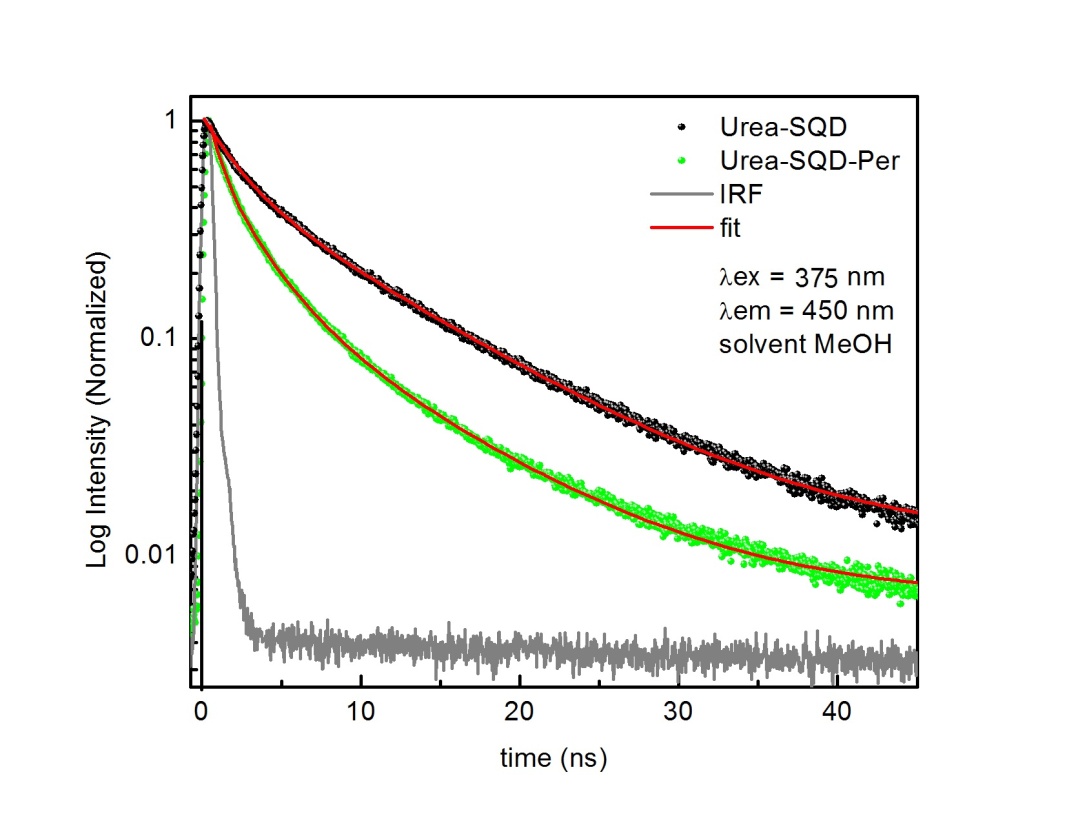
**

SI 6. Kinetic traces of Urea-SQD and Urea-SQD-Per collected at room temperature in methanol (λ_em_ and λ_ex_ indicated on graph; IRF ≈ 250 ps).

**
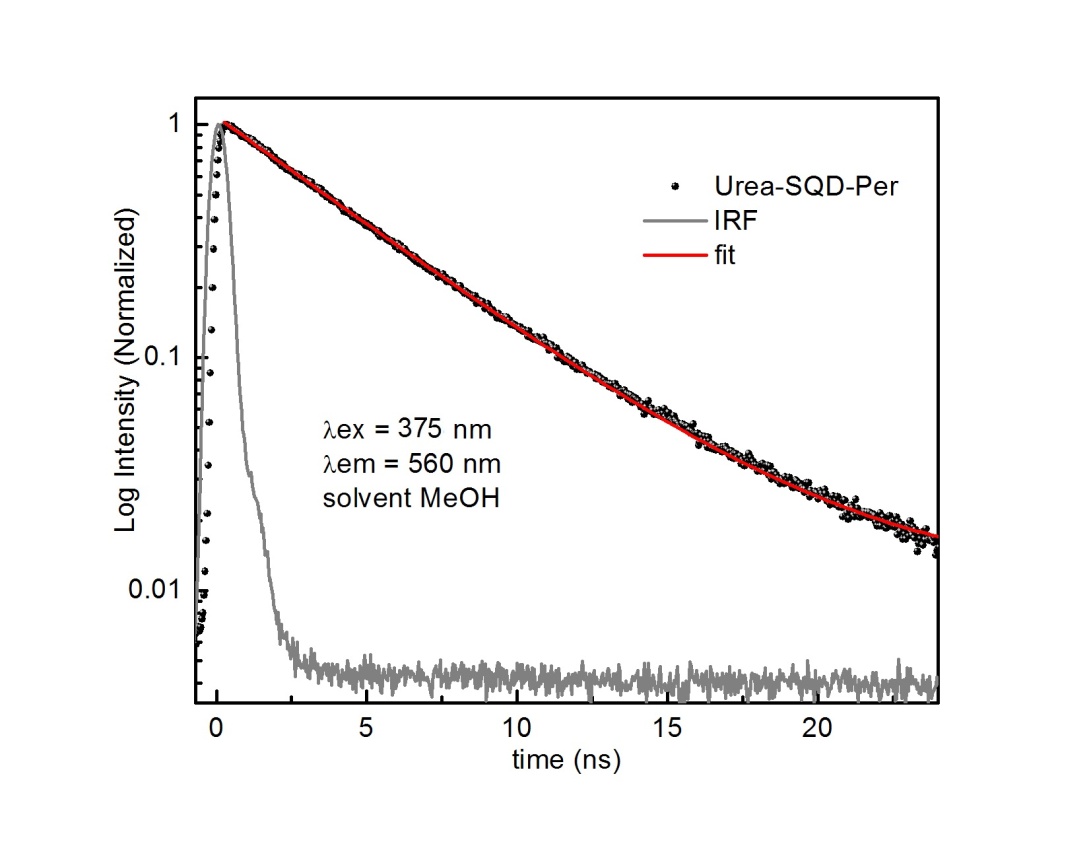
**

SI 7. Kinetic traces of Urea-SQD-Per collected at room temperature in methanol (λ_em_ and λ_ex_ indicated on graph; IRF ≈ 250 ps).

**
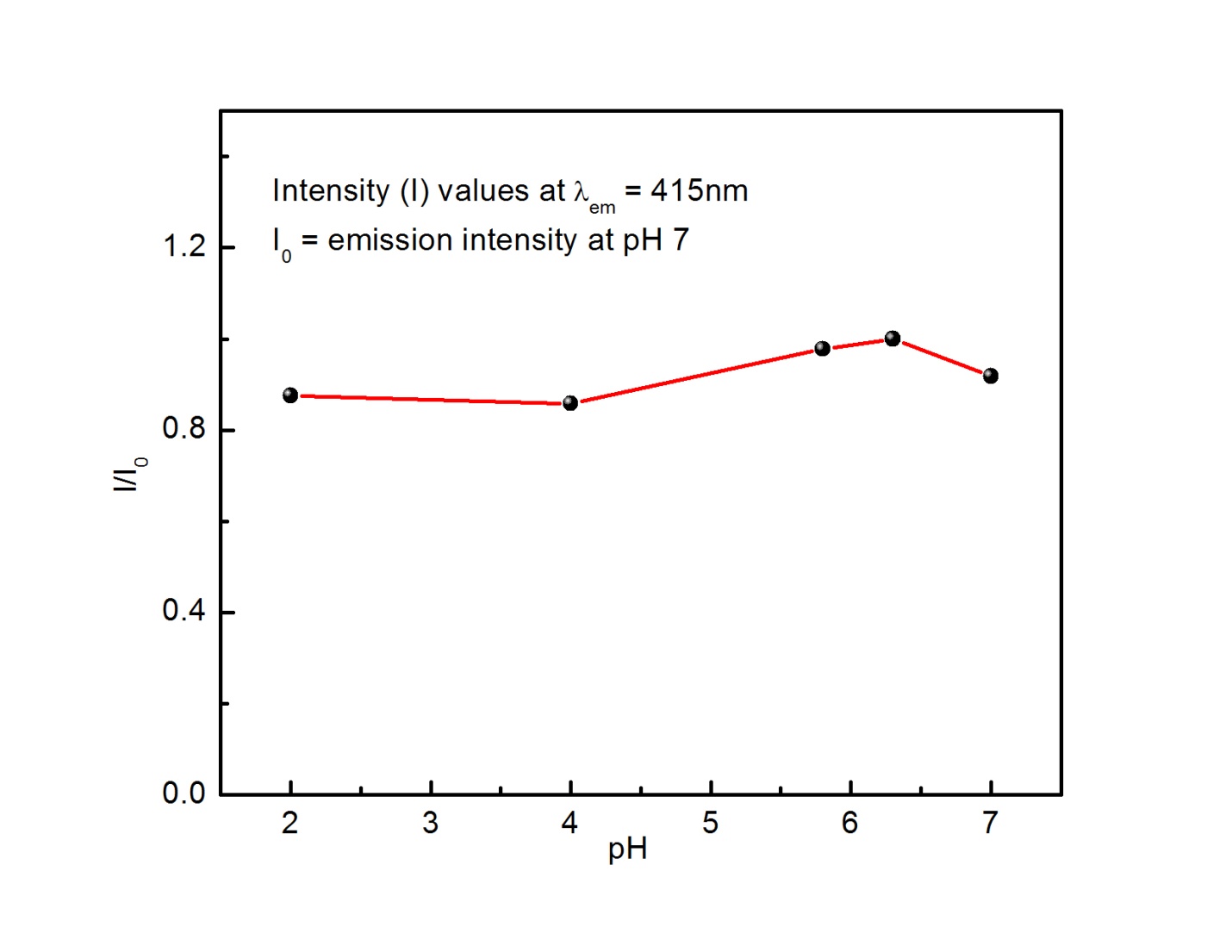
**

SI 8. Relative emission intensity as a function of pH change for Am-SQD-Per aqueous solutionusing λ_ex_ = 330 nm.


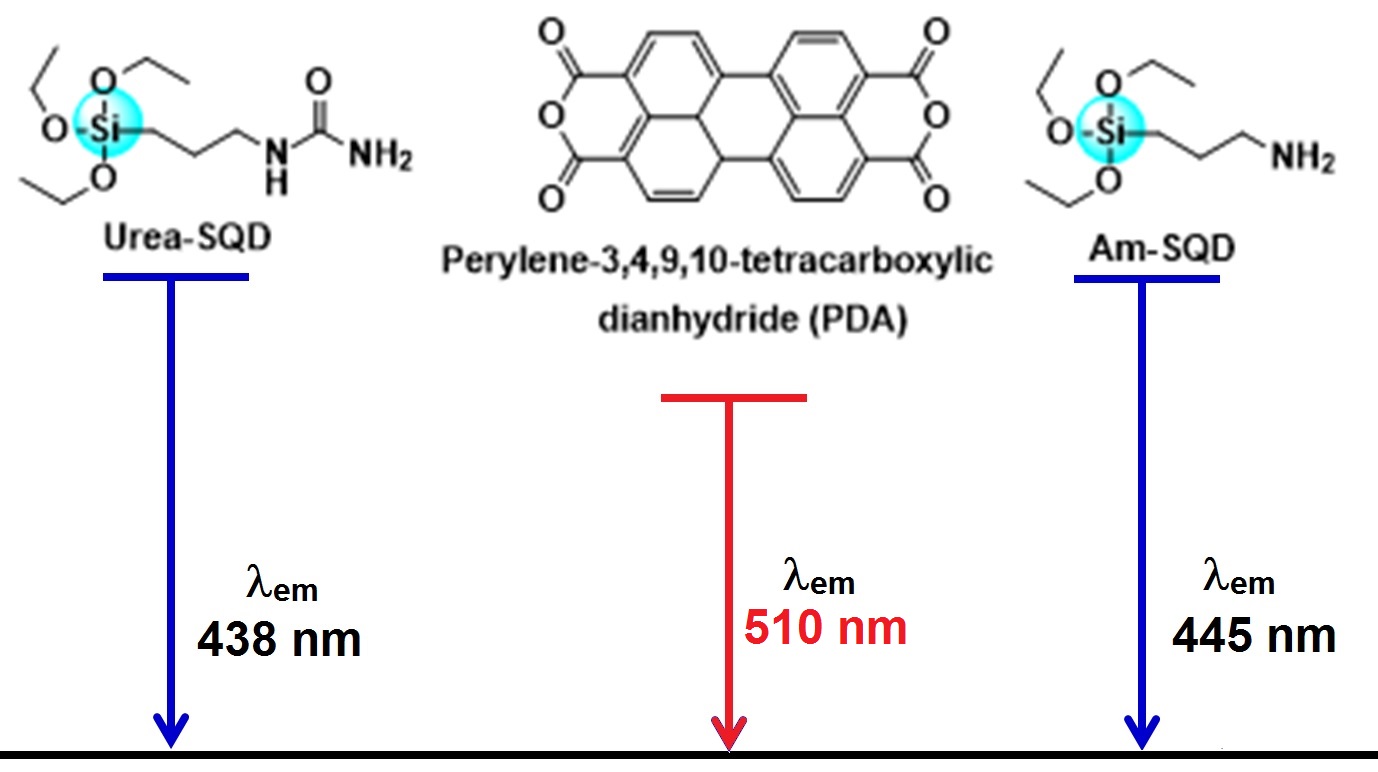


**SI 9.** Relative energy level diagram as predicted from the emission wavelength collected at room temperature.


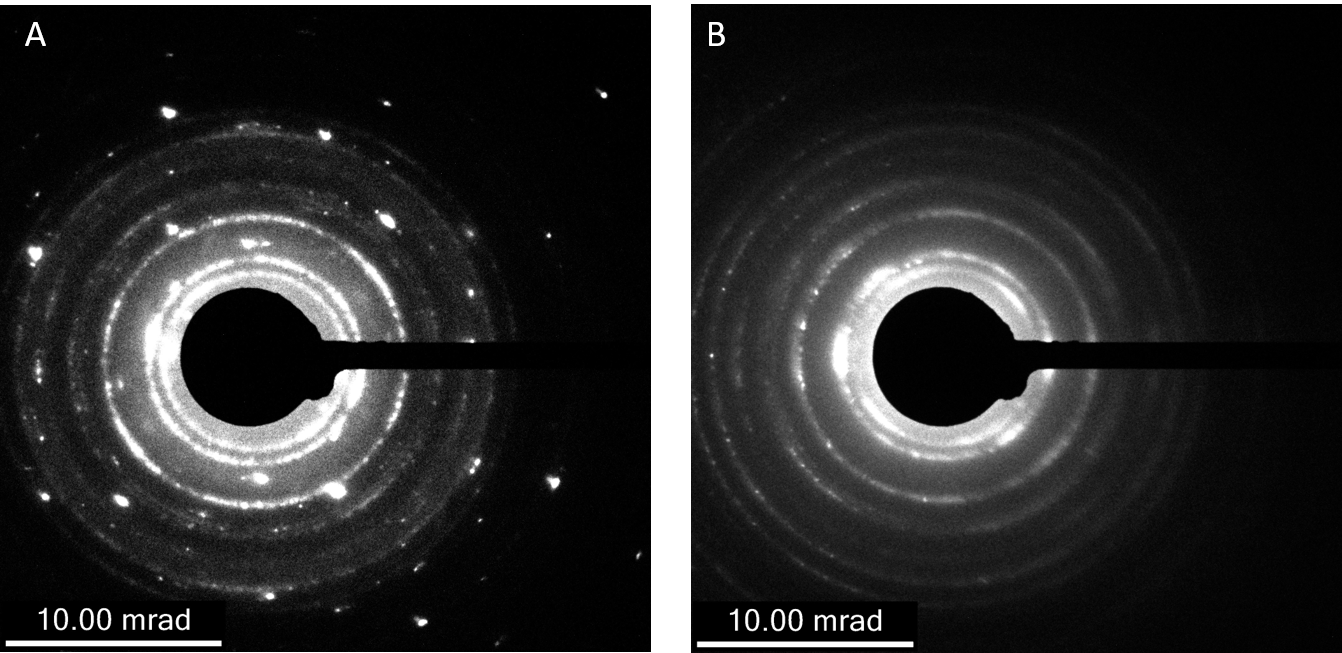


**SI 10.** SAED pattern of Am-SQD-Per (A) and Urea-SQD-Per (B).
